# *In vivo* Selection of Imipenem Resistance Among Ceftazidime-Avibactam-Resistant, Imipenem-Susceptible *Klebsiella pneumoniae* Isolate With KPC-33 Carbapenemase

**DOI:** 10.3389/fmicb.2021.727946

**Published:** 2021-09-23

**Authors:** Chunlei Wang, Jiankang Zhao, Zhibo Liu, Aihua Sun, Lingxiao Sun, Binbin Li, Binghuai Lu, Yingmei Liu, Bin Cao

**Affiliations:** ^1^Laboratory of Clinical Microbiology and Infectious Diseases, China-Japan Friendship Hospital, Department of Pulmonary and Critical Care Medicine, Beijing, China; ^2^Centre for Respiratory Diseases, China-Japan Friendship Hospital, Department of Pulmonary and Critical Care Medicine, Beijing, China; ^3^Chinese Academy of Medical Science, Clinical Research Center of Respiratory Diseases, Institute of Respiratory Medicine, Beijing, China; ^4^Clinical Center for Pulmonary Infections, Capital Medical University, Tsinghua University-Peking University Joint Center for Life Sciences, Beijing, China

**Keywords:** *Klebsiella pneumoniae* carbapenemase, ceftazidime-avibactam resistance, whole genome sequencing, KPC-33, KPC-producing *Klebsiella pneumoniae*

## Abstract

We describe *in vivo* evolution of carbapenem and ceftazidime-avibactam resistance by analyzing four longitudinal *Klebsiella pneumoniae* clinical isolates from a patient with pneumonia following antimicrobial treatment. The patient had fever, cough associated with expectoration, and new infiltration was found on the chest CT. Antimicrobial susceptibility was determined, and whole genome sequencing (WGS) was performed to investigate its dynamic change of resistance phenotype. Population analysis profile was performed to investigate the population of *Klebsiella pneumoniae*. The infection started with a KPC-2-producing *K. pneumoniae* (ZRKP01, ceftazidime-avibactam-S/carbapenem-R). Then, after ceftazidime-avibactam treatment, the strain switched to D179Y mutant that is KPC-33 (ZRKP02, ceftazidime-avibactam-R/carbapenem-S), which restored carbapenem susceptibility. However, the restored carbapenem susceptibility *in vivo* was not stable and the subsequent use of imipenem against KPC-33-producing *K. pneumoniae* infection resulted in a reversion of KPC-2 producers (ZRKP03 and ZRKP04, ceftazidime-avibactam-S/carbapenem-R). Genetic analysis demonstrated that all four *K. pneumoniae* isolates belonged to sequence type 11and had identical capsular polysaccharide (KL47), identical porin genes, and same plasmid replicon types. Phylogenetic analysis indicated that four *K. pneumoniae* isolates showed a high degree of relatedness. Single nucleotide polymorphisms analysis indicated that the number of mutations observed in the KPC-33 isolate was more than in the wild-type KPC-2 isolates and the four KPC-Kp isolates evolved from a longitudinal evolution of *K. pneumoniae* harboring *bla*_KPC-2_ gene. This is the first report to observe the *in vivo* evolution of wild-type KPC-2 to KPC-33 and then the reversion to its original wild-type KPC-2. Through WGS, we demonstrated the role of selective pressure of antibiotic in the mutation and reversion of *bla*_KPC_ genes, which leading to the dynamic change of KPC enzymes and the dynamic emergence of resistance to ceftazidime-avibactam and carbapenems.

**Statement:** Recently, studies reported the emergence of ceftazidime-avibactam-resistant strains. The KPC mutations mediating ceftazidime-avibactam resistance are generally associated with the restoration of carbapenem susceptibility. However, clinical significance of this observation is unclear. In this manuscript, we demonstrate the role of selective pressure of antibiotic in the mutation and reversion of *bla*_KPC_ genes, which leading to the dynamic change of KPC enzymes and the dynamic emergence of resistance to ceftazidime-avibactam and carbapenems. To the best of our knowledge, this is the first report to observe the *in vivo* evolution of wild-type KPC-2 to KPC-33 and then the reversion to its original wild-type KPC-2. It should be noted that understanding the clinical significance of this observation is of critical importance, and reversion to carbapenem susceptibility would not imply a potential role for carbapenems monotherapy. We hope our study will draw attention to clinicians, so that this agent can be used most effectively for the longest period of time.

## Introduction

Ceftazidime-avibactam is a β-lactam/β-lactamase inhibitor combination that was approved for the treatment of complicated intra-abdominal infections, complicated urinary tract infections, hospital-acquired pneumonia, and ventilator-associated pneumonia (Falcone and Paterson, 2016). The agent is highly active against class A β-lactamases including *Klebsiella pneumoniae* carbapenemases, class C β-lactamases, and OXA-48 carbapenemase, but not metallo-β-lactamases such as NDM, VIM, and IMP (van Duin and Bonomo, 2016). Despite a limited use of ceftazidime-avibactam at a worldwide scale, ceftazidime-avibactam resistance has been reported either in patient with no history of ceftazidime-avibactam therapy (Humphries, 2015) or in patient after short periods of ceftazidime-avibactam exposure (Shields et al., 2016). Resistance to ceftazidime-avibactam has been linked to specific mutations in the *bla*_KPC_ gene (Shields et al., 2017a), specific mutations in the *bla*_CTX-M_ gene (Both et al., 2017), porin deficiency combined with high ceftazidime hydrolysis (Shen et al., 2017; Galani et al., 2019), porin inactivation with or without increased expression of the *bla*_KPC_ gene (Humphries and Hemarajata, 2017; Coppi et al., 2020), or transposition of KPC with porin deficiency (Nelson et al., 2017). The mechanism most often associated with the emergence of ceftazidime-avibactam resistance after treatment has been observed to be mutations in the *bla*_KPC_ genes encoding for KPC enzymes, such as L169P, A177E, D179Y, D179N, V240G, Y241H, T243M, and H274N mutations in KPC (Shields et al., 2017a; Giddins et al., 2018; Hemarajata and Humphries, 2019; Sun et al., 2020). These mutational changes in KPC that emerged *in vivo* are often associated with fully or partially reversion to carbapenem susceptibility (Haidar et al., 2017; Shields et al., 2017a; Giddins et al., 2018). However, clinical significance of this observation is unclear, since subsequent exposure to carbapenems can restore resistance to them *in vitro* passage experiments (Shields et al., 2017b). In the present study, we observed the *in vivo* evolution of KPC-2 to KPC-33 and then the reversion to KPC-2, which leading to the dynamic emergence of resistance to ceftazidime-avibactam and carbapenems.

## Materials and Methods

### Bacterial Strains and Susceptibility Testing

The patient underwent a routine culture of sputum or bronchoalveolar lavage fluid (BALF) over a 3-month hospitalization. A total of 19 respiratory tract specimens from the patient were collected, including 12 sputum and 7 bronchoalveolar lavage. Four of these specimens yield *K. pneumoniae*. Isolates were identified as *K. pneumoniae* by MALDI-TOF MS (Bruker Daltonics, Billerica, MA, United States), and antimicrobial susceptibility testing (AST) was performed using the VITEK-2 compact system (bioMerieux, Marcy-l’Etoile, France). AST was further performed by means of broth microdilution, which was performed and interpreted according to the guidelines established by the Clinical and Laboratory Standards Institute, United States (CLSI, 2019). Avibactam was tested at a fixed concentration of 4mg/l in combination with increasing concentrations of ceftazidime.

### DNA Sequencing, *de novo* Assembly, and Annotation

For whole genome sequencing (WGS), the genomic DNA of four isolates was subjected to both short- and long-read massively parallel sequencing. Short-read sequencing was performed on the Illumina HiSeq 2,500 sequencing platform (Illumina, San Diego, CA), and long-read sequencing was performed using the Oxford Nanopore MinION platform (Oxford Nanopore, Oxford, United Kingdom). Raw reads were filtered to remove low-quality sequences and adaptors. *De novo* assembly was conducted using SPAdes Genome Assembler v3.13.1 (Bankevich et al., 2012; Nurk et al., 2013) and Unicycler (Wick et al., 2013). Gene prediction was performed using Prokka 1.12 (Seemann, 2014). Antimicrobial resistance genes and plasmid replicon analysis were performed using ResFinder and PlasmidFinder tools *via* the CGE server.^1^ Prophages and insertion sequences in the genome were identified with PHASTER (Amdt et al., 2016) and ISsaga (Varani et al., 2011), respectively. Mutations present in isolates were analyzed as follows: Sequence reads were mapped to the reference genome of strain ZRKP01 using Bowtie 2 (Langmead and Salzberg, 2012), and single nucleotide polymorphisms (SNPs) were identified with SAMtools (Li et al., 2009) and Genome Analysis Toolkit (Mckenna et al., 2010). All SNPs were manually checked. Subsequently, we constructed a maximum likelihood phylogenic tree by using RAxML (Stamatakis, 2014) with general time reversible model of nucleotide substitution and a Gamma distribution of rate heterogeneity.

### Population Analysis Profile (PAP) of Four KPC-Producing *Klebsiella pneumoniae* (KPC-Kp) Isolates

To investigate the presence of meropenem and ceftazidime/avibactam heteroresistance, population analysis profiles were determined by spiral plating 50-μl aliquots of the starting bacterial cell suspension on Mueller-Hinton agar plates without or with various concentrations of meropenem and ceftazidime-avibactam (0, 0.125, 0.25, 0.5, 1, 2, 4, 8, 16, 32, 64, 128, 256, and 512mg/l) as previously described (Li et al., 2006; Gaibani et al., 2018). Avibactam was tested at a fixed concentration of 4μg/ml in combination with increasing concentrations of ceftazidime. The analysis was conducted in three replicates, and carbapenem-susceptible *K. pneumoniae* ATCC 25922 was used as the control strain. After 48h of incubation at 35°C, the number of colonies was counted.

## Results

### Patient-Clinical Context

**Figure 1:**
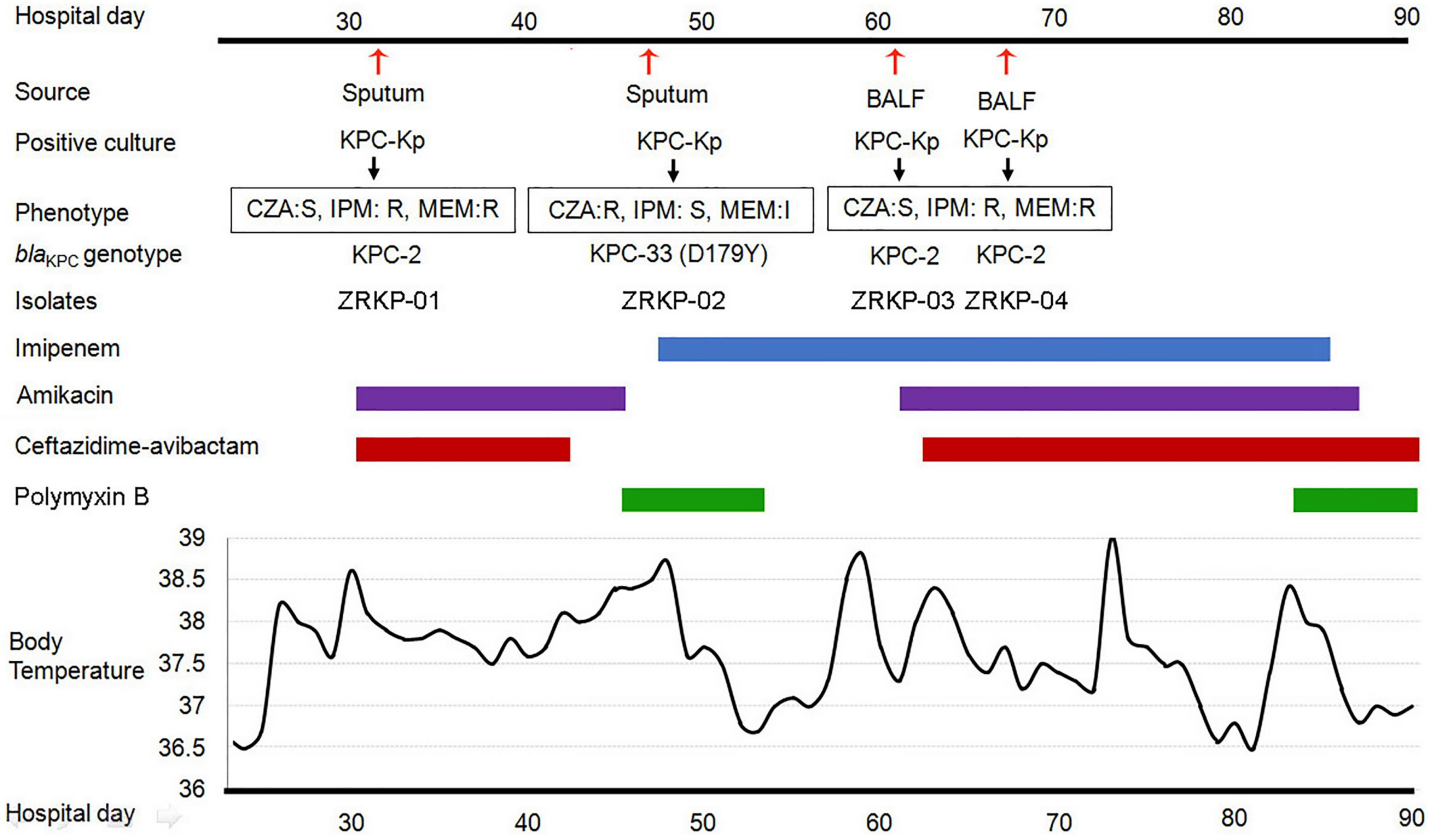
History of the *K. pneumoniae* isolation and clinical antimicrobial treatment course of a patient with KPC-Kp infection. Blue bar represents the imipenem, purple bars represent the amikacin, red bars represent ceftazidime-avibactam, and green bars represent polymyxin B. The red arrows show the isolation times of *K. pneumoniae* (isolate ZRKP01, ZRKP02, ZRKP03, and ZRKP04). CZA, Ceftazidime-avibactam; IMP, Imipenem; MEM, Meropenem; BALF, bronchoalveolar lavage fluid.

The patient was a 75-year-old man who had been diagnosed with *Mycobacterium avium-intracellulare* complex lung disease 2years earlier and had been treated with antimicrobial therapies since then. Antimicrobial therapy included ethambutol, clarithromycin, moxifloxacin, and linezolid. The patient had been admitted to the hospital several times over the past 2years for the lung disease. However, the infective process and radiological findings persisted despite the repeated courses of antimicrobial therapies. *M. avium-intracellulare* in sputum samples also persisted. He was admitted to the department of pulmonary and critical care medicine, China-Japan Friendship Hospital due to a fever. During hospitalization, patient was treated with clarithromycin (0.5g every day; 0.25g qn) and ethambutol (750mg every day) for *M. avium-intracellulare* complex lung disease. On day 32 post-admission, under therapy with ceftazidime-avibactam plus amikacin for 3days, a carbapenem-resistant KPC-producing *K. pneumoniae* (isolate ZRKP01) was recovered from a sputum. On hospital day 47, under therapy with ceftazidime-avibactam plus amikacin for 12days, a ceftazidime-avibactam-resistant but imipenem-susceptible isolate was recovered from sputum (isolate ZRKP02, exhibited a phenotype compatible with an ESBL producer). The antibacterial therapy was changed to imipenem plus polymyxin B. 15days after imipenem therapy, bronchoalveolar cultures yielded a carbapenem-resistant *K. pneumoniae* (isolate ZRKP03 and isolate ZRKP04) on hospital day 61 and again on day 67, respectively, which prompted reinitiation of amikacin plus ceftazidime-avibactam with the continued addition of imipenem. After imipenem plus ceftazidime-avibactam plus amikacin was started, five respiratory tract specimens were collected between days 72 and 90 that did not yield microorganisms. However, the patient developed respiratory failure and died. The patient’s antibiotic treatment course and the timeline of the *K. pneumoniae* isolates acquired are summarized in Figure 1.

### Emergence of an Apparent Phenotypic Change of *K. pneumoniae* Isolates During Infection

During the course of illness, four serial *K. pneumoniae* isolates were cultured from respiratory tract specimens on hospital 32, 47, 61, and 67days, respectively. Testing of susceptibility to ceftazidime-avibactam and carbapenems showed three distinct susceptibility phenotypes as shown in Table 1. Phenotype 1 (isolate ZRKP01), collected at the baseline (under therapy with ceftazidime-avibactam for 3days), was susceptible to ceftazidime-avibactam (MIC, 0.5mg/l) and resistant to carbapenem (IMP, MIC 32mg/l; MEM, MIC 128mg/l). Phenotype 2 (isolate ZRKP02), collected following 12days ceftazidime-avibactam, displayed ceftazidime-avibactam resistance (MIC, 64mg/l) and restored susceptibility to carbapenem (IMP, MIC 0.06mg/l; MEM, MIC 2mg/l). Of note, phenotype 3 (isolate ZRKP03), collected after 15days imipenem plus 6days polymyxin B, reverted to carbapenem resistance (IMP, MIC 128mg/l; MEM, MIC 512mg/l) and restored susceptibility to ceftazidime-avibactam (MIC, 2mg/l; although with elevated MIC compared with baseline isolate ZRKP01). Phenotype 3 (isolate ZRKP04), collected during imipenem, ceftazidime-avibactam plus amikacin combination therapy, was susceptible to ceftazidime-avibactam (MIC, 2mg/l) and resistant to carbapenem (IMP, MIC 64mg/l; MEM, MIC 256mg/l; a lower MIC compared with ZRKP03). These isolates displayed in an apparent phenotypic change from carbapenem-resistant to susceptible and then reverted to resistant for carbapenem, while the phenotypic change from ceftazidime-avibactam-susceptible to resistant then reverted to susceptible for ceftazidime-avibactam.

**Table 1:** Summary of antimicrobial susceptibility testing for four *K. pneumoniae* isolates recovered from a patient with pneumonia.

Isolates No.	MIC (mg/L)								
	CZA	IMP	MEM	CAZ	ATM	FEP	TZP	TGC	CST
ZRKP01 (KPC-2)	0.5	32	128	>=64	>=64	>=64	>=128	1	0.5
ZRKP02 (KPC-33)	64	0.06	2	>=64	>=64	>=64	>=128	1	0.5
ZRKP03 (KPC-2)	2	128	512	>=64	>=64	>=64	>=128	1	1
ZRKP04 (KPC-2)	2	64	256	>=64	>=64	>=64	>=128	1	1

*CZA, Ceftazidime-avibactam; CAZ, Ceftazidime; IMP, Imipenem; MEM, Meropenem; ATM, Aztreonam; FEP, Cefepime; TZP, Piperacillin/tazobactam; TGC, Tigecycline; CST, Colistin*.

### Genome Comparison of Four KPC-Kp Isolates

**Figure 2:**
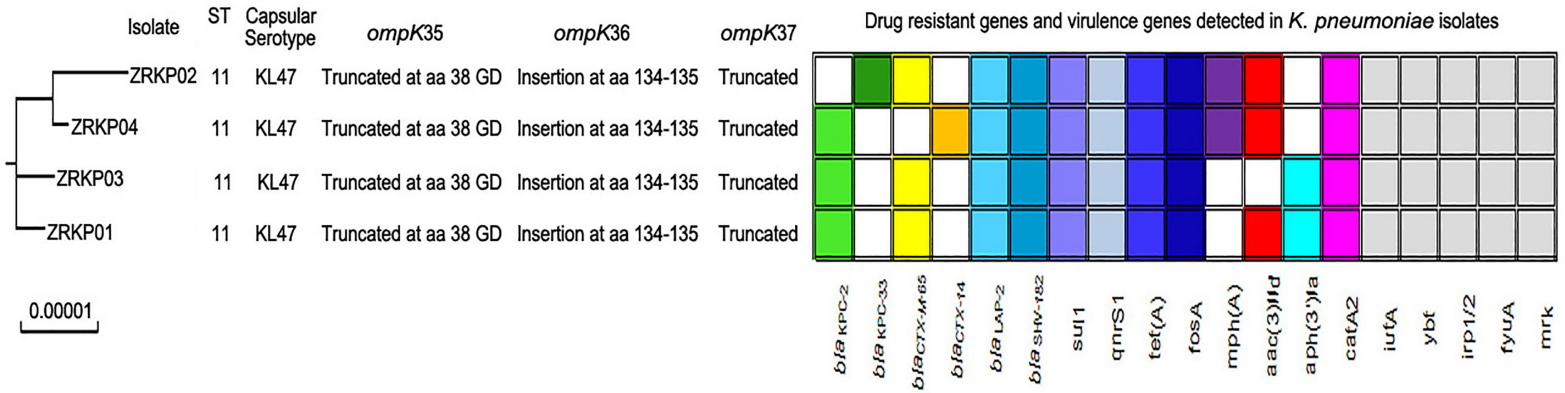
Molecular characteristic of four *K. pneumoniae* isolates.

Genetic analysis demonstrated that all four KPC-Kp isolates belonged to sequence type (ST11) and had identical capsular polysaccharide (KL47; *wzc*: 47, *wzi*: 209), a common CRE species in China. All the four isolates had genes encoding KPC, SHV-182, and CTX-M β-lactamases. Other acquired resistance genes justifying the resistance phenotype and virulence genes are depicted in Figure 2. Analysis of outer membrane porin genes demonstrated that all the four isolates had a mutated *ompK*35 gene encoding truncated porin (premature stop codon at amino acid position 88) and had a mutated *ompK*36 gene encoding a porin OmpK36 with a GD insertion at amino acid position 134–135.

**Figure 3:**
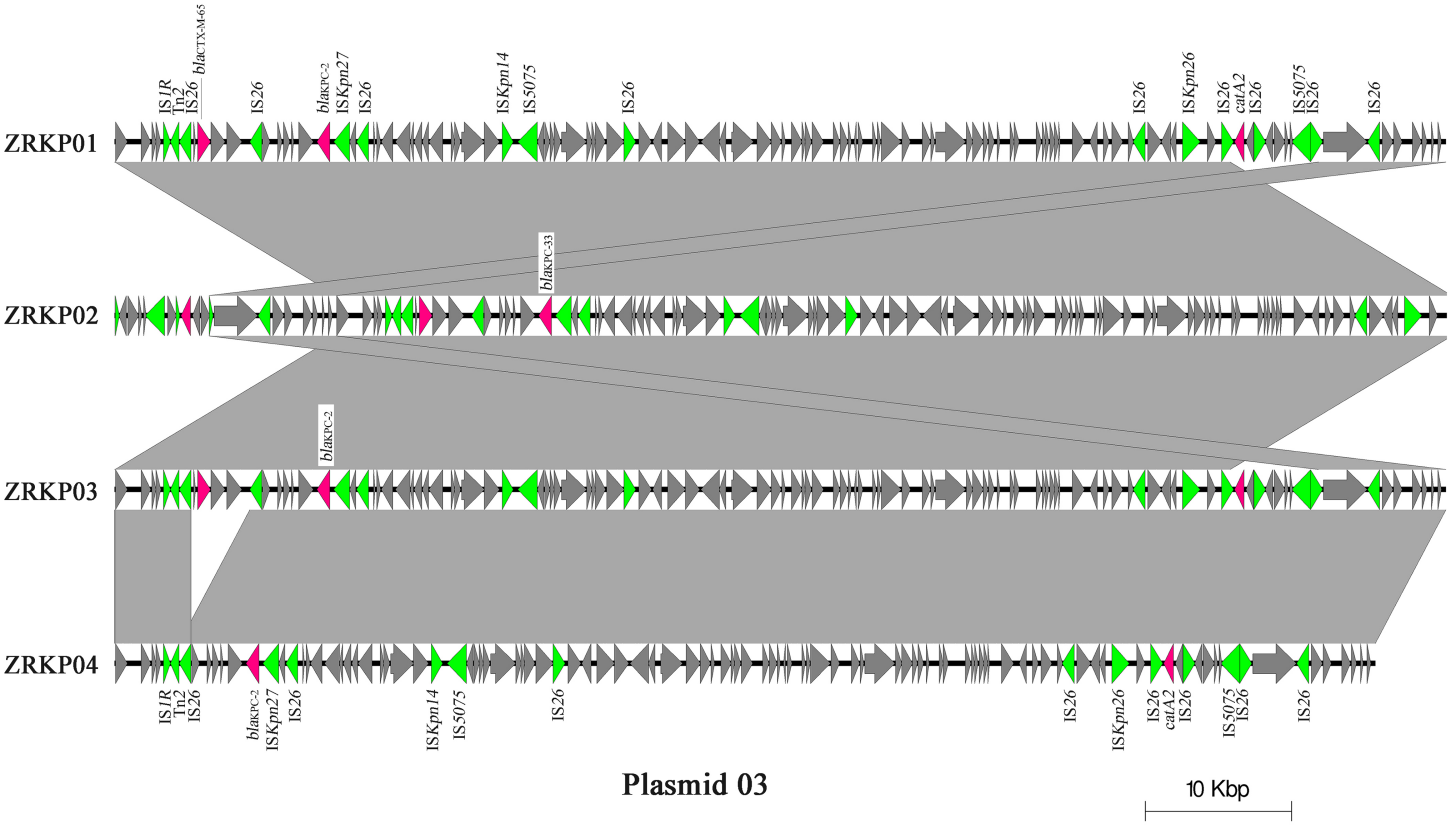
Major structural features of plasmid 03 identified in the four *K. pneumoniae* isolates. Light grey shading denotes shared regions of homology, and open reading frames (ORFs) are portrayed by arrows. Pink arrows represent antibiotic resistant genes, and green arrows represent transposon-related genes and insertion sequences. The plasmid sequence of sample ZRKP02 was reverse-complemented to get a better presentation.

The genomic features of assemblies are presented in Table 2. The chromosome lengths of ZRKP01, ZRKP02, ZRKP03, and ZRKP04 were 5.435 Mbp, 5.438 Mbp, 5.434 Mbp, and 5.436 Mbp, respectively, similar in length to other *K. pneumoniae* genomes in public databases (range, 5.3–5.6 Mbp). They had an average G + C content of 56.9% and carried 25 ribosomal RNA genes and 85 transfer RNA genes. The four *K. pneumoniae* isolates harbored five similar plasmids referred as plasmid 01, plasmid 02, plasmid 03, plasmid 04, and plasmid 05. Plasmid content analysis showed that all four KPC-Kp isolates shared the same plasmid replicon types as shown in Table 2. Furthermore, the fusion was observed of IncFII and IncFIB in plasmid 01 and IncFII and IncR in plasmid 03. The plasmid 03 lengths of ZRKP01, ZRKP02, ZRKP03, and ZRKP04 were 91,097, 91,502, 91,097, and 86,253 base pairs, respectively. The *bla*_KPC_ gene was located in plasmid 03 as shown in Figure 3. Ceftazidime-avibactam-susceptible isolates (ZRKP01, ZRKP03, and ZRKP4) carried *bla*_KPC-2_, whereas ceftazidime-avibactam-resistant isolate (ZRKP02) carried mutant *bla*_KPC-2_ (*bla*_KPC-33_) encoding for KPC enzymes. Deep examination of reads aligning to the *bla*_KPC-2_ gene demonstrated that 94% of aligned reads of the ZRKP01 isolate displayed the wild-type (KPC-2) and 6% displayed D179Y mutation (KPC-33). We did not find coexistence of wild-type KPC-2 and mutation D179Y (KPC-33) in the WGS data of the remaining three *K. pneumoniae* isolates (ZRKP02-ZRKP04). Corresponding to the phenotype change, the *K. pneumoniae* isolates displayed an genotype change from wild-type KPC-2 to variant KPC-2 (KPC-33) and then reversion to its original wild-type KPC-2.

**Table 2:** Genomic features of four *Klebsiella pneumoniae* ST11 isolates.

Feature	ZRKP01	ZRKP02	ZRKP03	ZRKP04
G+C content, %	56.98	56.98	56.99	56.99
Plasmids, no.	5	5	5	5
Size, base pairs				
Chromosome	5,435,561	5,438,841	5,434,420	5,436,990
Plasmid 01	239,469	220,761	205,940	238,333
Plasmid 02	110,529	110,529	110,529	110,529
Plasmid 03	91,097	91,502	91,097	86,253
Plasmid 04	10,060	10,060	10,060	10,060
Plasmid 05	5,596	5,596	5,596	5,596
*Plasmid (Inc)*				
Plasmid 01	IncFIB, IncFII	IncFIB, IncFII	IncFIB, IncFII	IncFIB, IncFII
Plasmid 02	IncFIB	IncFIB	IncFIB	IncFIB
Plasmid 03	IncFII, IncR	IncFII, IncR	IncFII, IncR	IncFII, IncR
Plasmid 04	ColRNAI	ColRNAI	ColRNAI	ColRNAI
Plasmid 05	NA	NA	NA	NA
Genes, no.	5,186	5,189	5,180	5,182
CDS, no.	5,076	5,079	5,070	5,072
Ribosomal RNA genes, no.	25	25	25	25
Transfer RNA genes, no.	85	85	85	85
Prophages, no.	15	14	14	14
IS elements, no.	50	53	49	51
Chromosome IS family (no.)	*IS*1 (4), *IS*3 (9), *IS*5 (25), *IS*6 (1), *IS*481 (1), *IS*1182 (1), *IS*1380 (3), *IS*NCY (6)	*IS*1 (3), *IS*3 (9), *IS*5 (27), *IS*6 (1), *IS*110 (2), *IS*481 (1), *IS*1182 (1), *IS*1380 (3), *IS*NCY (6)	*IS*1 (3), *IS*3 (9), *IS*5 (25), *IS*6 (1), *IS*481 (1), *IS*1182 (1), *IS*1380 (3), *IS*NCY (6)	*IS*1 (3), *IS*3 (9), *IS*5 (26), *IS*6 (1), *IS*110 (2), *IS*481 (1), *IS*1182 (1), *IS*1380 (3), *IS*NCY (6)

*CDS, coding DNA sequences; IS, insertion sequence*.

**Figure 4:**
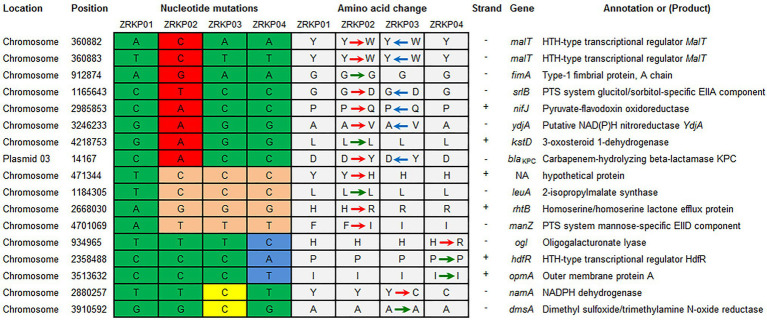
Summary of mutations present in four *K. pneumoniae* isolates and their effects on annotated coding sequences. The mutations in ZRKP 02 (KPC-33) isolate accounted for 9 of 11 total nonsynonymous mutations. Six of these targets (2 *malt, srlB*, *nifJ*, *ydjA*, *and bla*_KPC-33_) reverted to its original wild-type, including wild-type KPC-2 confer resistance to carbapenem in ZRKP 03 and ZRKP04 isolates. The remaining 3 mutant genes (*rhtB*, *manZ*, and a hypothetical protein) showed clonal succession in ZRKP 03 and ZRKP04 isolates. Amino acid abbreviations follow the standard one-letter code. Red arrows represent the nonsynonymous substitutions, green arrows represent the synonymous substitutions, and blue arrows represent the reversion of the nonsynonymous substitutions.

Using the genome assembly of *K. pneumoniae* isolate (ZRKP01) as a reference, we identified 17 substitutions among *K. pneumoniae* isolates comprising 6 synonymous and 11 nonsynonymous substitutions. SNP analysis revealed that the isolate ZRKP02 (KPC-33) evolved by up to 12 SNPs/genome, the ZRKP03 evolved by 6 SNPs/genome, and the ZRKP04 evolved by 7 SNPs/genome over the patient’s hospitalization. Numbers and positions of SNPs for each pairwise comparison of isolates are shown in Figure 4. In our analysis of the evolutionary process in this case, we found that the number of mutations observed in the ZRKP02 (KPC-33) isolate was more than in the wild-type KPC-2 isolates (ZRKP01, ZRKP03, and ZRKP04). These mutations in ZRKP02 isolate accounted for 9 of 11 total nonsynonymous mutations. Of note, 6 of these targets (2 *malt, srlB*, *nifJ*, *ydjA*, *and bla*_KPC-33_) reverted to its original wild-type on further imipenem treatment; in particular, wild-type KPC-2 confer resistance to carbapenem. On the other hand, the remaining 3 mutant genes (*rhtB*, *manZ*, and a hypothetical protein) showed clonal succession (Figure 4) Therefore, it demonstrated that the four KPC-Kp isolates evolved from a longitudinal evolution of *K. pneumoniae* harboring *bla*_KPC-2_ gene.

### PAP of Four KPC-Kp Isolates

**Figure 5:**
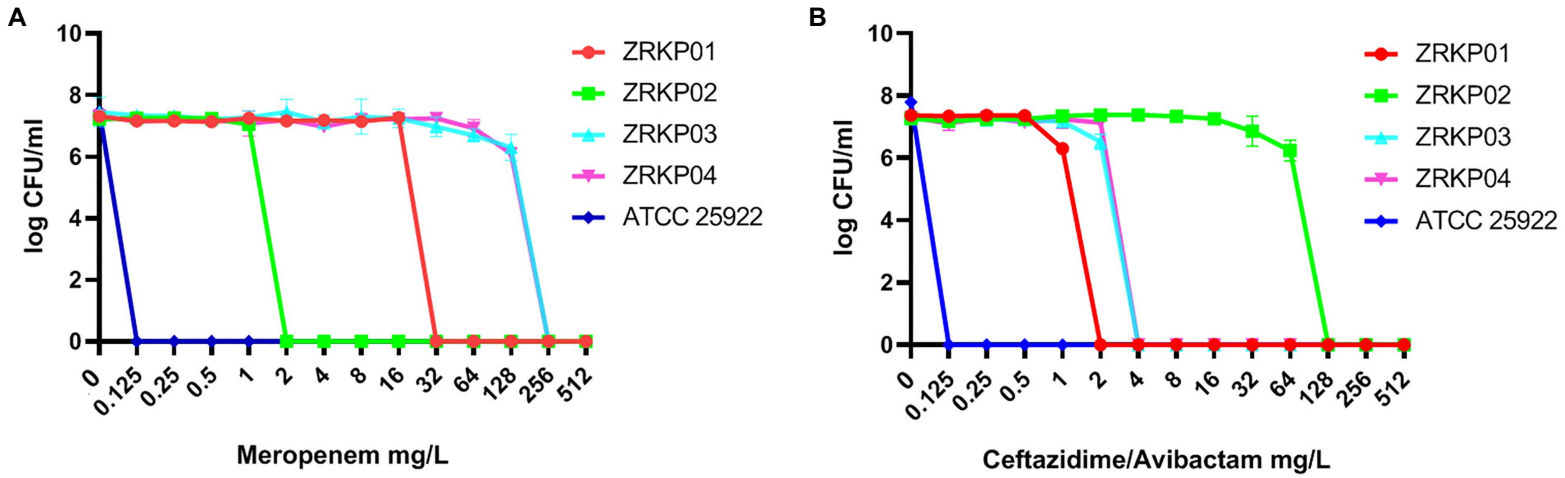
Population analysis profile (PAP) curves of the four KPC-producing *K. pneumoniae* clinical isolates. PAP at different concentrations of meropenem **(A)** and ceftazidime-avibactam **(B)**.

PAP curves of the four KPC-Kp isolates are shown in Figure 5. Population analysis showed that both the meropenem-resistant subpopulation and ceftazidime-avibactam-resistant subpopulation were not found in all four isolates (CRKP01-CRKP04).

## Discussion

Here, we report the dynamic emergence of resistance to ceftazidime-avibactam and carbapenems in KPC-Kp during antimicrobial therapy. Using WGS of four longitudinal clinical isolates, we directly documented clonal succession of the *bla*_KPC-2_ conferring carbapenems and *bla*_KPC-33_ conferring ceftazidime-avibactam resistance.

The patient described in this study was intermittently exposed to 40days of ceftazidime-avibactam. After 12days ceftazidime-avibactam plus amikacin treatment, ceftazidime-avibactam-resistant KPC-33 (KPC-2 D179Y variant) producing *K. pneumoniae* strain appeared, as indicated from previous study (Shields et al., 2016; Haidar et al., 2017; Shields et al., 2017a; Giddins et al., 2018; Hemarajata and Humphries, 2019; Sun et al., 2020). Of note, KPC-33 producing *K. pneumoniae* (ceftazidime-avibactam-resistant) isolate was unable to preserve the resistance phenotype without the selective pressure of ceftazidime-avibactam and it reverted to susceptible phenotype on further selective pressure of imipenem. Importantly, the restored carbapenem susceptibility was not stable and subsequently reverted to its original carbapenem resistance phenotype on the selective pressure of imipenem. It should be noted that understanding the clinical significance of this observation is of critical importance, and reversion to carbapenem susceptibility would not imply a potential role for carbapenems monotherapy. In addition, the efficacy of dual ceftazidime-avibactam and carbapenem therapy in these settings is unclear. More studies are needed to establish the precise role of carbapenems in treating such infections and the durability of restored carbapenem susceptibility *in vivo*.

Similarly, the *in vitro* reversion of *bla*_KPC-3_ mutations by *K. pneumoniae* isolates was recently described by Shields et al., 2017b; isolates with D179Y substitutions in KPC-3 when exposed to meropenem, *bla*_KPC-3_ mutations reverted to wild type, were replaced by new mutations, or were retained (Shields et al., 2017b). This *in vitro* reversion of *bla*_KPC-3_ mutation was also observed by Göttig et al. from Germany (Göttig et al., 2019). They found that isolate with D179Y substitution in KPC-3, *bla*_KPC-3_ mutation reverted to wild type, and demonstrated the isolate with mutational change in KPC-3 under selection pressure are associated with ceftazidime-avibactam resistance, while imipenem resistance was solely due to reversion of KPC-3 D179Y to wild-type KPC-3. To the best of our knowledge, this is the first report to observe the *in vivo* evolution of wild-type KPC-2 to KPC-33 and then the reversion to its original wild-type KPC-2.

Mechanisms of horizontal gene spread among *K. pneumoniae* often considered to be the main mediators of antibiotic resistance, such as acquisition and loss of antibiotic resistance genes as previously described (Villa et al., 2013; Simner et al., 2018). However, mutational resistance also has primary clinical importance when considering resistance to particular antibiotics, especially to carbapenems and ceftazidime-avibactam (Haidar et al., 2017; Shields et al., 2017a; Giddins et al., 2018; Hemarajata and Humphries, 2019; Sun et al., 2020). In the present study, genetic analysis demonstrated that all four *K. pneumoniae* isolates belonged to sequence type 11, had identical capsular polysaccharide (KL47), identical porin genes, and same plasmid replicon types. Phylogenetic analysis indicated that four *K. pneumoniae* isolates showed a high degree of relatedness. PAPs showed no co-mixed infection population. These results demonstrate the four KPC-Kp isolates evolved from a longitudinal evolution of *K. pneumoniae* harboring *bla*_KPC-2_ gene. Our result indicated that the variant KPC can be relatively unstable, which may result in KPC enzymes dynamic change through mutations or reversions to alter their spectra of activity under different selection pressure.

In the course of ceftazidime-avibactam treatment, the dynamic change of resistance to ceftazidime-avibactam and carbapenems has been documented in KPC-Kp clinical isolates (Gaibani et al., 2018; Sun et al., 2020). However, little is known regarding the mechanisms of dynamic change. Gaibani et al. found that two different subpopulations harboring wild-type and mutant *bla*_KPC-3_ coexisting in the same KPC-Kp clinical isolate and the coexistence of different variants within a single isolate determining a hybrid phenotype resulting in resistance to both carbapenems and ceftazidime/avibactam (Gaibani et al., 2018). Heteroresistance to ceftazidime-avibactam was also observed in our previous study (Sun et al., 2020). We described coexistence of wild-type KPC-2(29.4%) and mutation D179Y (KPC-33, 71.6%) within a single isolate, which may contribute resistance to both carbapenems and ceftazidime-avibactam. In the present study, through WGS, we demonstrated the role of selective pressure of antibiotic in the mutation and reversion of *bla*_KPC_ genes, which leading to the dynamic change of KPC enzymes and the dynamic emergence of resistance to ceftazidime-avibactam and carbapenems.

It should be noted that *K pneumoniae* with mutant *bla*_KPC_ may be identified as ESBL producers (rather than KPC producers), if carbapenemase screening is triggered by elevated carbapenem MICs (Shields et al., 2017a). Targeted sequencing of *bla*_KPC_ of isolates may prove to be powerful tools for rapidly identifying loss or restored activity of ceftazidime-avibactam and carbapenems, respectively.

## Conclusion

This study described the plasticity and speed of evolutionary changes in KPC-Kp strains and stressed the importance of mutations and reversion of *bla*_KPC_ genes in dynamic change of resistance to ceftazidime-avibactam and carbapenems.

## Data Availability Statement

The raw data supporting the conclusions of this article will be made available by the authors to any qualified researcher. The assembly genome sequences have been deposited on NCBI with BioProject ‘PRJNA613645’.

## Ethics Statement

Permission for using the information in the medical records of the patients and the K. pneumoniae isolates for research purposes was granted by the ethical committee of China-Japan Friendship Hospital (2019-164-K113).

## Author Contributions

BC and YL conceived or designed the work. ZL, AS, and BL collected the clinical data. BL and LS collected the laboratory data and performed the tests. CW and JZ analyzed and interpreted the data. CW, JZ, YL, and BC drafted the manuscript. All authors contributed to the article and approved the final version of the manuscript to be published.

## Funding

This work was financially supported by the National Key R&D Program of China (2017YFC1309301 and 2017YFC1309300), CAMS Innovation Fund for Medical Sciences (CIFMS 2018-I2M-1-003), and National Science Grant for Distinguished Young Scholars (81425001/H0104) for BC.

## Conflict of Interest

The authors declare that the research was conducted in the absence of any commercial or financial relationships that could be construed as a potential conflict of interest.

## Publisher’s Note

All claims expressed in this article are solely those of the authors and do not necessarily represent those of their affiliated organizations, or those of the publisher, the editors and the reviewers. Any product that may be evaluated in this article, or claim that may be made by its manufacturer, is not guaranteed or endorsed by the publisher.

## Footnotes

^1^ https://cge.cbs.dtu.dk/services/
